# Supplementary material for: ERLIN1/2 scaffolds bridge TMUB1 and RNF170 and restrict cholesterol esterification to regulate the secretory pathway
Source: Life Sci Alliance. 2024 May 24;7(8):e202402620. doi: 10.26508/lsa.202402620 (PMC11116810; doi:10.26508/lsa.202402620)
Supplement: Supplementary file 1 [file LSA-2024-02620_SdataF1_F2_F3_FS1_FS2_FS3.pdf]

Figure 1B Western Blot raw data
Unspecific bands are marked with an asterisk (*)

Loading order: WT-DKO-DKO[+E1/E2]

ERLIN1

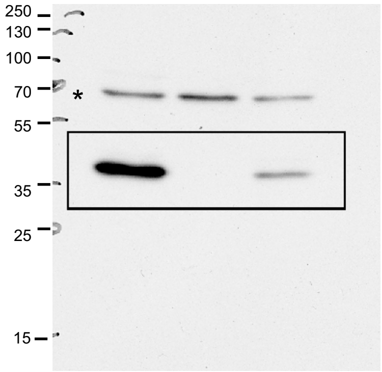

ERLIN2

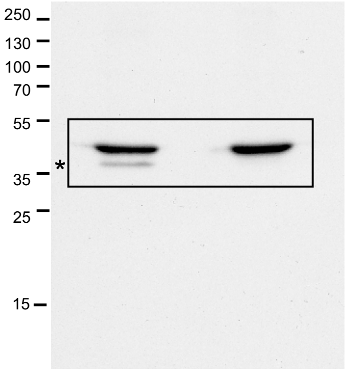

GAPDH
ERLIN1

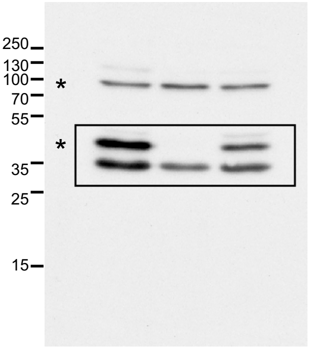

GAPDH
ERLIN2

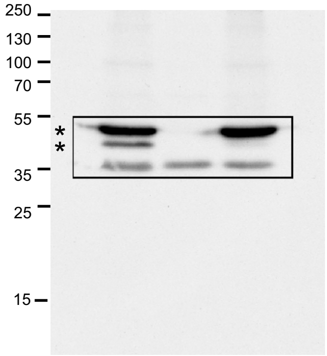

Figure 1D Western Blot raw data

Fractions

Loading order: Input-I-II-III-IV-V

↑

DRMs

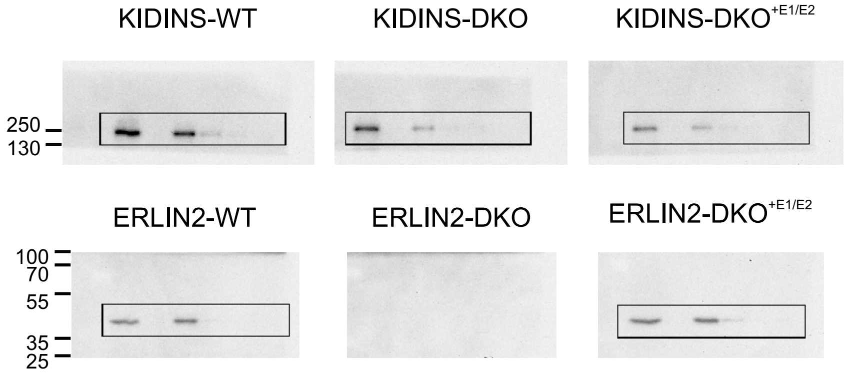

Membranes were cut at 100 Kda: the upper part was incubated with anti-KIDINS antibody and the lower with anti-ERLIN2 antibody

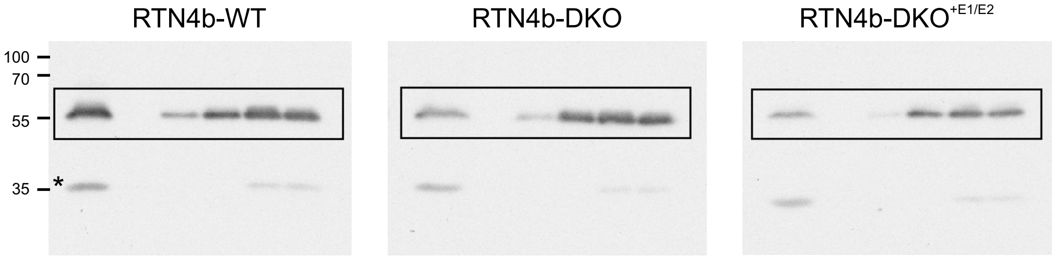

Membranes were previously incubated with GAPDH (not the one shown in the manuscript)

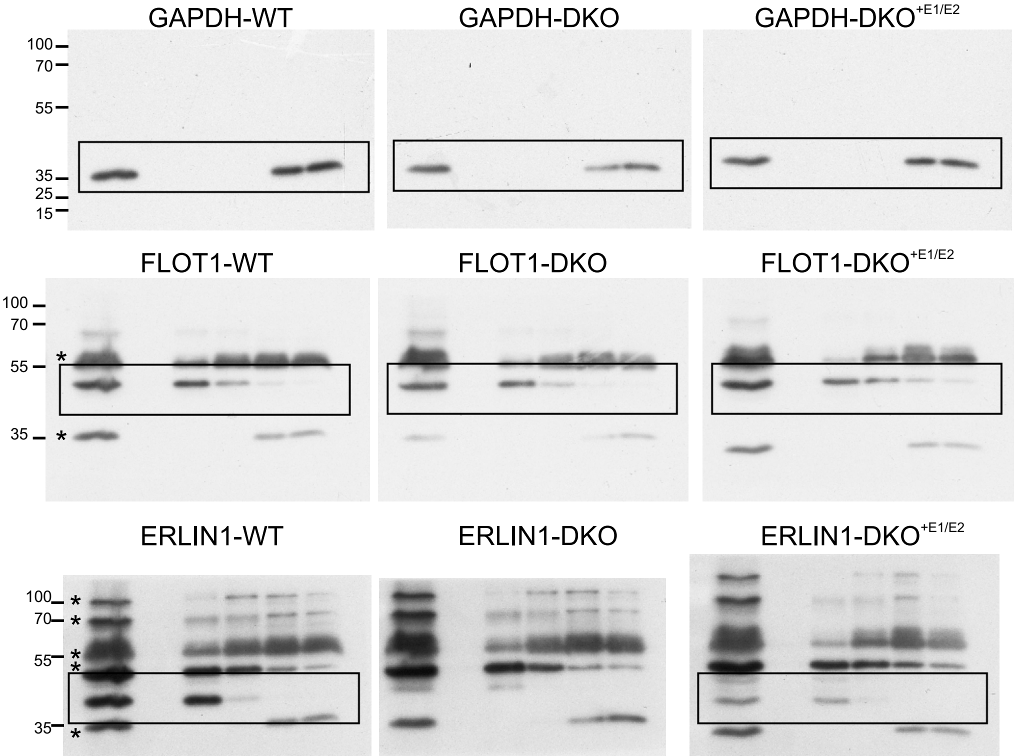

Membranes were incubated sequentially with anti-GAPDH, anti-RTN4b (not used the one used in the manuscript), anti-FLOT1 and anti-ERLIN1

Figure 2B Western Blot raw data ERLIN2-IP

Input — IP — Flow Through

Loading order:WT-DKO-WT-DKO-WT-DKO

ERLIN2-WB

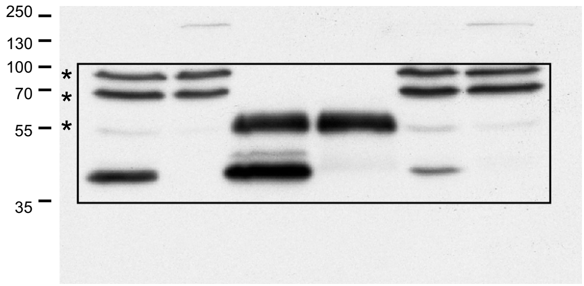

TMUB1-WB

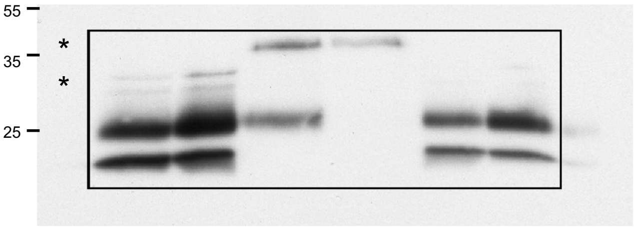

Figure 2C Western Blot raw data TMUB1-IP

IP — Flow Through

Loading order: Input-TMUB1-IgG-TMUB1-IgG

TMUB1-WB

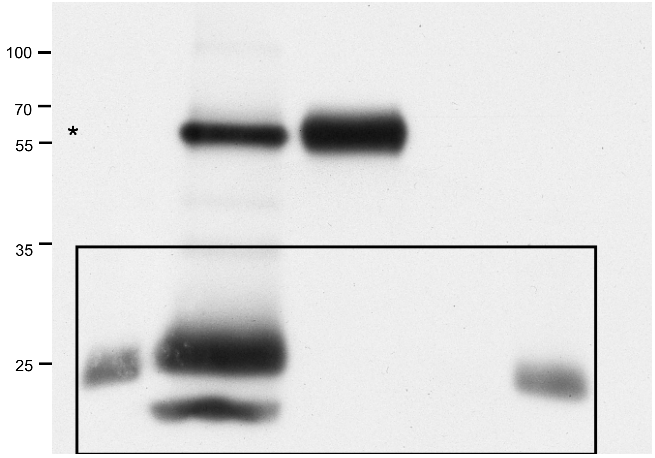

ERLIN2-WB

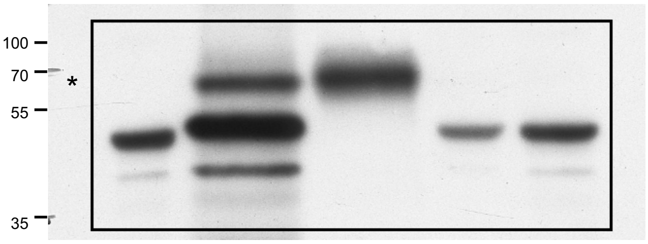

Figure 2D Western Blot raw data TMUB1-DRMs

Fractions

Loading order: Input-I-II-III-IV-V

DRMs

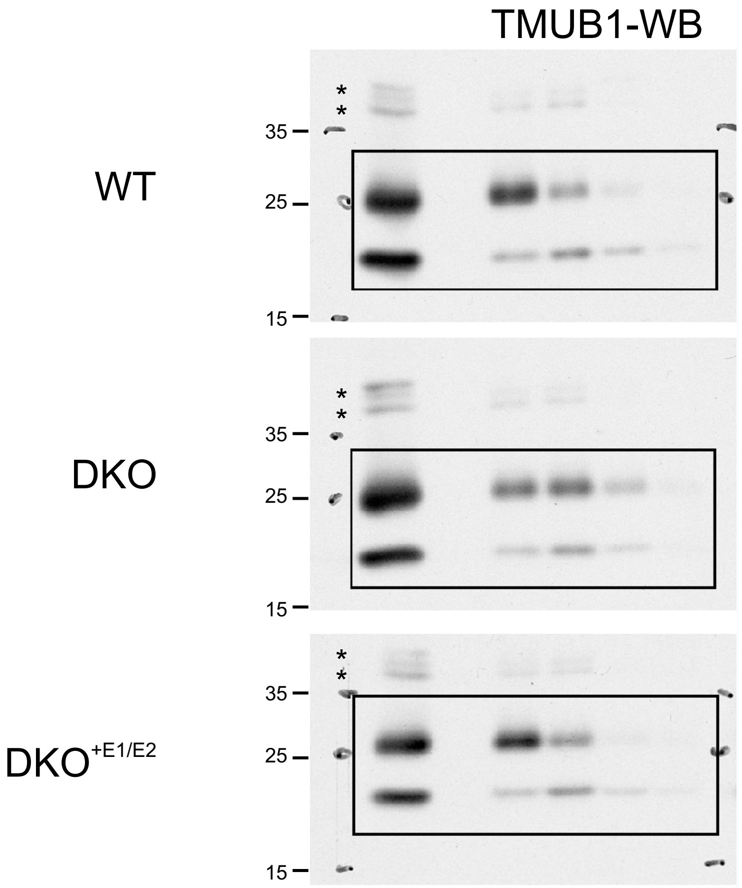

Figure 3D Western Blot raw data

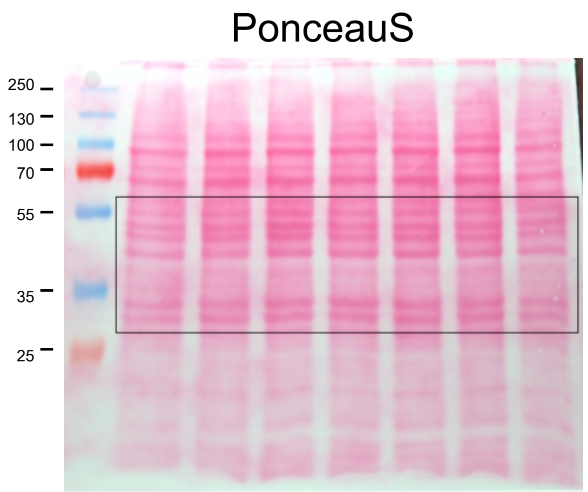

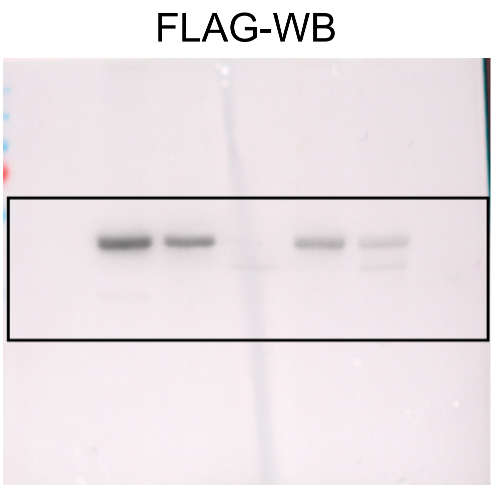

Loading order: Empty-WT-R36K-G48V-H50Y-R36K&H50Y-Triple

Figure S1 Western Blot raw data

Fractions

Loading order: Input-I-II-III-IV-V

DRMs

TMEM259

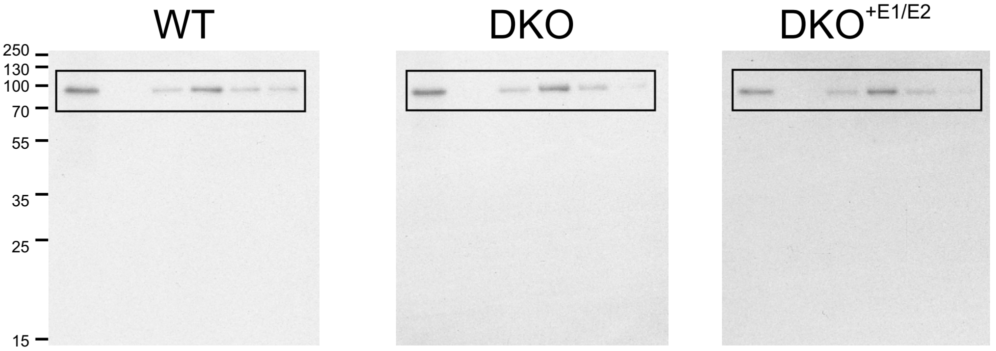

VDAC2

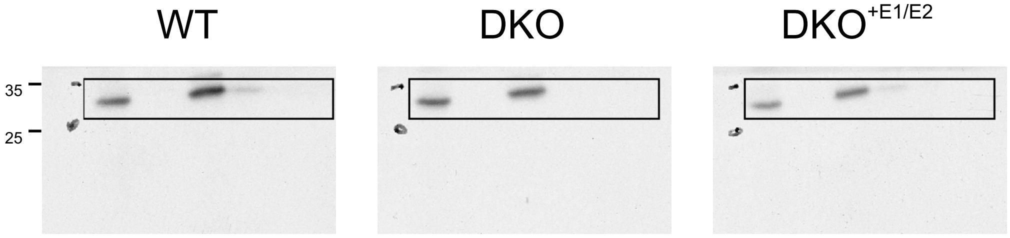

Membranes were cut above 35 Kda and lower part was incubated with VDAC2

PMP70

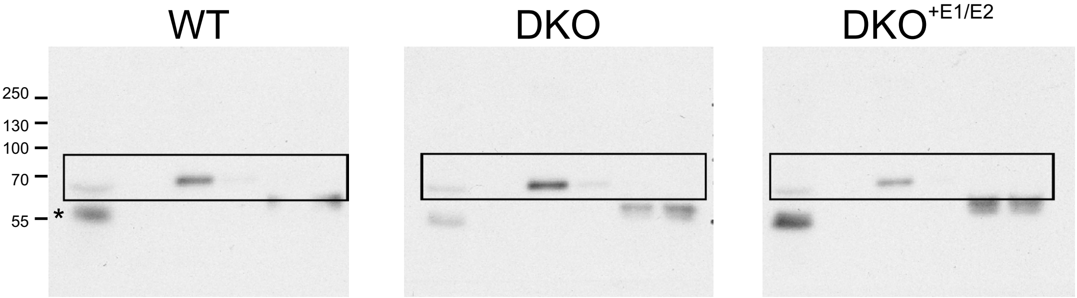

TMUB2

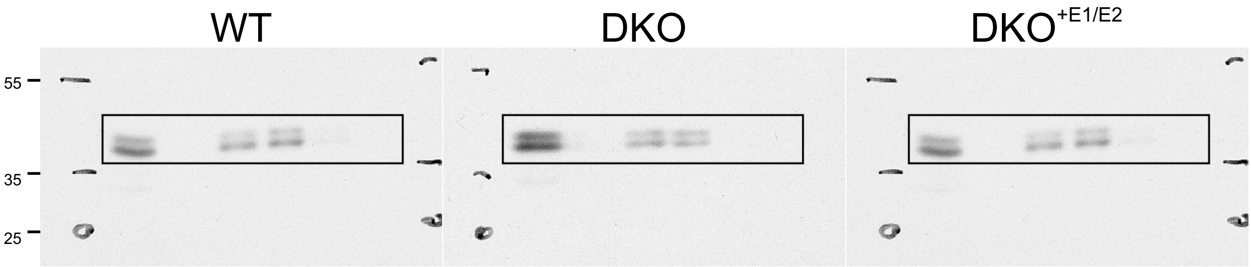

Membranes were cut at 70 Kda and lower part was incubated with TMUB2

Figure S2A Western Blot raw data

TMUB1-L-KO

Loading order: WT-Clone_I-Clone_II

TMUB1-WB Actin-WB

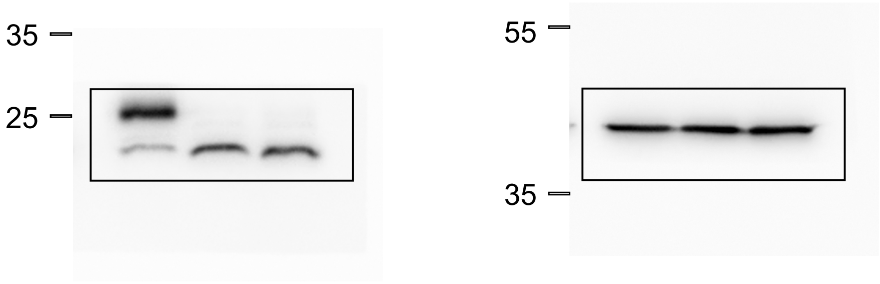

Figure S2E Western Blot raw data

TMUB1
siRNA

Loading order: Lipofectamine_CTR-siRNA_CTR-I-II-III-Mix
Lipofectamine_CTR is not used in the manuscript

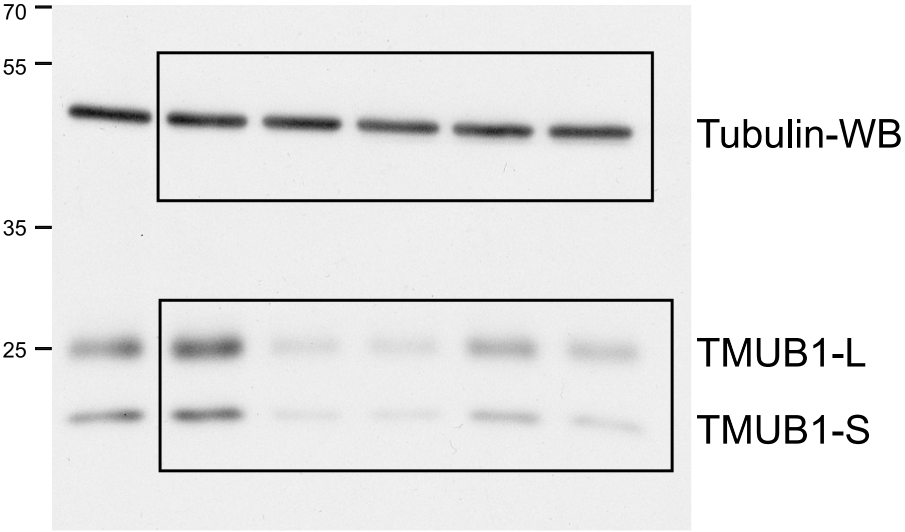

The membrane was cut at 35 Kda, the upper part was incubated with anti-tubulin antibody and the lower part with anti-TMUB1 antibody. The two sections were developed together.

Figure S3A Western Blot raw data

Loading order: WT-DKO-DKO$^{+E1/E2}$-WT-DKO-DKO$^{+E1/E2}$-WT-DKO-DKO$^{+E1/E2}$

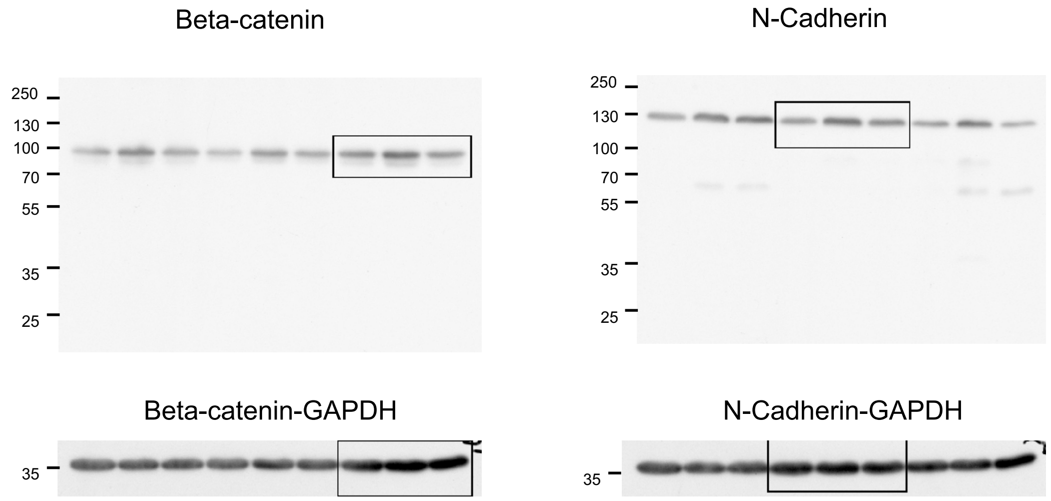

The membranes were cut at 55 Kda and lower part was incubated with anti-GAPDH antibody
